# Mechanism of BDNF Modulation in GABAergic Synaptic Transmission in Healthy and Disease Brains

**DOI:** 10.3389/fncel.2018.00273

**Published:** 2018-08-28

**Authors:** Christophe Porcher, Igor Medina, Jean-Luc Gaiarsa

**Affiliations:** ^1^Aix Marseille University, Marseille, France; ^2^Institut National de la Santé et de la Recherche Médicale (INSERM) U901, Marseille, France; ^3^Institut de Neurobiologie de la Méditerranée (INMED), Marseille, France

**Keywords:** BDNF, TrkB, p75^NTR^, GABA receptors, KCC2

## Abstract

In the mature healthy mammalian neuronal networks, γ-aminobutyric acid (GABA) mediates synaptic inhibition by acting on GABA_A_ and GABA_B_ receptors (GABA_A_R, GABA_B_R). In immature networks and during numerous pathological conditions the strength of GABAergic synaptic inhibition is much less pronounced. In these neurons the activation of GABA_A_R produces paradoxical depolarizing action that favors neuronal network excitation. The depolarizing action of GABA_A_R is a consequence of deregulated chloride ion homeostasis. In addition to depolarizing action of GABA_A_R, the GABA_B_R mediated inhibition is also less efficient. One of the key molecules regulating the GABAergic synaptic transmission is the brain derived neurotrophic factor (BDNF). BDNF and its precursor proBDNF, can be released in an activity-dependent manner. Mature BDNF operates via its cognate receptors tropomyosin related kinase B (TrkB) whereas proBDNF binds the p75 neurotrophin receptor (p75^NTR^). In this review article, we discuss recent finding illuminating how mBDNF-TrkB and proBDNF-p75^NTR^ signaling pathways regulate GABA related neurotransmission under physiological conditions and during epilepsy.

## Introduction

A striking trait of early GABAergic transmission is that activation of γ-aminobutyric acid (GABA_A_) receptors (GABA_A_Rs) causes membrane depolarization and Ca^2+^ influx in immature neurons (Ben-Ari et al., 1989, 2007; Ganguly et al., 2001). During this critical period, depolarizing GABA_A_R activity plays a major role in neuronal network construction (Ben-Ari et al., 2007; Wang and Kriegstein, 2008; Sernagor et al., 2010). Given this fundamental role it comes as no surprise that flawed GABAergic transmission is implicated in an array of brain disorders such as epilepsy (Ben-Ari and Holmes, 2005), autism spectrum disorder (ASD), Rett syndrome (Kuzirian and Paradis, 2011), schizophrenia (Lewis et al., 2005; Charych et al., 2009; Mueller et al., 2015) and major depressive disorder (Sanacora et al., 1999; Brambilla et al., 2003). GABAergic development relies heavily on brain derived neurotrophic factor (BDNF; Hong et al., 2008; Gottmann et al., 2009; Sakata et al., 2009; Kuzirian and Paradis, 2011), one of the most crucial regulator of synapse development and function in the developing and adult central nervous system (CNS; Lu et al., 2005; Cohen-Cory et al., 2010). BDNF can be secreted either as a precursor (proBDNF) or a mature form (mBDNF; Nagappan et al., 2009; Yang et al., 2009). ProBDNF and mBDNF modulate the efficacy of synaptic responses via the tropomyosin-related kinase receptor B (TrkB) and the p75 neurotrophin receptor (p75^NTR^), respectively (Lu et al., 2005). BDNF shapes the development of neuronal circuits, as well as the construction of inhibitory connections throughout life (Kovalchuk et al., 2004; Gubellini et al., 2005; Gottmann et al., 2009) and alterations in BDNF processing have been observed in diseases of the CNS, including schizophrenia, ASD and epilepsy (Binder et al., 2001; Carlino et al., 2011; Garcia et al., 2012). In this review article, we discuss the recent achievements in analysis of the development of GABAergic network with an emphasis on GABA and BDNF interplay. We particularly focus on ionotropic GABA_A_ or metabotropic GABA_B_ receptors activation in triggering the postsynaptic release of BDNF, which in turn regulates the maturation of GABAergic synapses. We then discuss how BDNF tunes up or down inhibitory transmission by acting on synthesis and trafficking of GABA_A_Rs and KCC2 chloride ion transporters at the cell membrane. Finally, we focus on epilepsy, a pathology that highlights the links between GABA and BDNF.

## BDNF and Inhibitory Strength of GABA_A_ Receptors

GABA_A_Rs are ionotropic receptors that allow the bidirectional flux of chloride ions across the neuronal membrane. The direction of Cl^−^ flux depends on [Cl^−^]_i_ and the membrane potential, whereas the intensity of the flux depends on the number of activated GABA_A_Rs. In mature healthy neurons the [Cl−]_i_ is close to 4 mM, and the reversal potential of the ion flux through GABA_A_Rs (EGABA_A_) is ~78–82 mV, close to the resting membrane potential (Tyzio et al., 2003; Khazipov et al., 2004). Hence, at rest, the activation of GABA_A_Rs produces no or, at the most, a weak (1–2 mV) hyperpolarization or depolarization. The activation of GABA_A_Rs during neuronal depolarization induced by the excitatory synapses allows massive Cl^−^ entry that provides strong hyperpolarizing force and effectively compensates or diminishes the strength of the excitatory signal. The increased [Cl^−^]_i_ is rapidly extruded by electroneutral neuron-specific potassium-chloride cotransporter KCC2 (Rivera et al., 1999). In immature neurons as well as in mature neurons during different pathologies (epilepsy (Cohen et al., 2002), acute trauma (Boulenguez et al., 2010), Rett syndrome (Banerjee et al., 2016), Down syndrome (Deidda et al., 2015), Huntington disease (Dargaei et al., 2018), ASD (Tyzio et al., 2014)) the activation of GABA_A_Rs produces neuron depolarization reflecting increased resting level of [Cl^−^]_i_. This Cl^−^-dependent depolarization facilitated the activation of the neuronal network and contributes to the formation of pathological patterns of network activities (Ben-Ari et al., 2007; Moore et al., 2017). Thus, the inhibitory strength of GABA_A_R mediated inhibition is determined by two complementary parameters: the amount of ion flux through opened GABA_A_Rs and the [Cl^−^]_i_. The mBDNF and proBDNF do regulate these two parameters.

## ProBDNF, mBDNF and GABA_A_R Interplay

Expression patterns of BDNF and proBDNF are developmentally regulated. ProBDNF expression levels increase during the first postnatal weeks while mature BDNF peaks at a later period (Yang et al., 2014; Menshanov et al., 2015; Winnubst et al., 2015). ProBDNF can be cleaved under physiological conditions depending mainly on neuronal activity generated in the developing neuronal networks (Lessmann and Brigadski, 2009; Nagappan et al., 2009; Langlois et al., 2013). For instance, theta burst stimulation triggers the co-release of proBDNF and the serine protease, tissue Plasminogen Activator (t-PA) which converts plasminogen to plasmin yielding to mature BDNF, whereas low-frequency stimulation increases the amounts of proBDNF in the extracellular space (Nagappan et al., 2009). Overexpression of proBDNF in proBDNF-HA/^+^ mice showed a decrease in dendritic arborization and spine density of hippocampal neurons as well as altered synaptic transmission (Yang et al., 2014). In developing rat hippocampal neurons, proBDNF/p75^NTR^ signaling has been reported to induces a long-lasting depression of GABA_A_R-mediated synaptic activity (Langlois et al., 2013), whereas endogenous BDNF/TrkB signaling is required for the induction of GABAergic long-term-potentiation (Gubellini et al., 2005).

**Figure 1:**
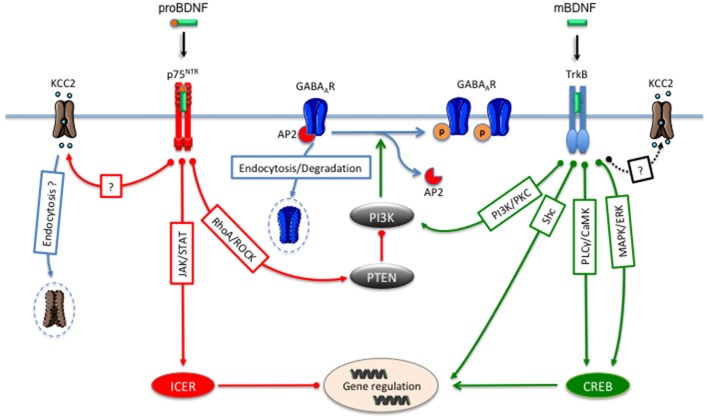
mBDNF/TrkB and proBDNF/p75^NTR^ signaling pathways regulate γ-aminobutyric acid (GABA) neurotransmission. Activation of TrkB receptors by mBDNF leads to an inhibition of GABA_A_R endocytosis and a consequent increase in the cell surface expression of these receptors through the PI 3-kinase and the PKC signaling pathway. At the transcriptional level, BDNF/TrkB signaling regulates GABA_A_R and KCC2 gene expression through the Shc, PLC/CaMK or MAP/ERK pathways. Activation of p75^NTR^ by proBDNF decreases GABA_A_Rs cell surface expression through the RhoA/ROCK/PTEN pathway that leads to the dephosphorylation of GABA_A_R and endocytosis and degradation of internalized receptors. At the transcriptional level, proBNDF/P75^NTR^ leads to the repression of GABA_A_R synthesis through the JAK2/STAT3/ICER pathway. The proBDNF/75^NTR^ signaling also decreases KCC2 expression.

In the cerebral cortex, BDNF/TrkB signaling controls the development of interneurons (Yuan et al., 2016) and the expression of the presynaptic GABA synthetic enzyme GAD65 (Sánchez-Huertas and Rico, 2011). In the cerebellum, BDNF promotes the formation of inhibitory synapses (Chen et al., 2016). Postsynaptically, BDNF and proBDNF are critical to control the GABA_A_Rs trafficking between synaptic sites and endosomal compartments. The cell membrane expression of GABA_A_Rs depends on their phosphorylation level (Nakamura et al., 2015). Thus, dephosphorylation of the GABA_A_R ß3 subunits triggers the association with the assembly polypeptide 2 (AP2) complex which leads to a clathrin-mediated internalization (Kittler et al., 2000; Nakamura et al., 2015). In fact, BDNF/TrkB signaling inhibits the internalization of GABA_A_Rs through activation of the phosphoinositide-3 kinase (PI-3 kinase) and PKC pathways (Figure 1). This ability of BDNF to modulate GABA_A_Rs endocytosis and activity is likely to occur due to an inhibition of their interaction with the protein phosphatase 2A complex (PP2A), a downstream target of PI-3 kinase (Jovanovic et al., 2004; Vasudevan et al., 2011). Inversely, application of proBDNF to cultured rat hippocampal neurons cause a reduction in GABAergic synaptic transmission by promoting dephosphorylation and internalization of GABA_A_R ß3 subunits through the RhoA–Rock–PTEN (phosphatase and tensin homolog) signaling cascade (Riffault et al., 2014). The underlying molecular mechanism of PTEN-mediated dephosphorylation and downregulation of GABA_A_Rs remains to be determined but may involve the inhibition of PI3-kinase activity and the subsequent upregulation of PP2A activity. Accordingly, PTEN activated by p75^NTR^ is a major negative regulator of the PI3-kinase signaling cascade (Song et al., 2010). Thus, the cell surface expression levels of GABA_A_Rs can be settled by the competition between mBDNF/TrkB and proBDNF/p75^NTR^ intracellular cascades on the PTEN/PI3-kinase-mediated activation of PP2A. After endocytosis, the proBDNF/p75^NTR^/Rho-ROCK pathway moved internalized GABA_A_Rs to late endosomes and finally to lysosomes for degradation (Riffault et al., 2014).

The BDNF may also be involved in GABA_A_Rs clustering at synaptic sites through the regulation of the main scaffolding protein gephyrin. Indeed, in immature rat hippocampal neuronal cultures BDNF enhanced the expression and clustering of gephyrin, which in turn leads to an increase in the density of GABA_A_Rs-gephyrin containing complexes at postsynaptic sites (González, 2014). Conversely, in cultured mouse amygdala neurons, rapid application of BDNF decreased the cell surface expression of GABA_A_Rs-gephyrin complexes whereas long-term treatment with BDNF elicits opposite effects (Mou et al., 2013). BDNF can exert different roles depending on the developmental stages (young vs. adult neurons) but also in function of the brain structures or according to the delivery mode (rapid vs. long-term treatment). These opposing responses of BDNF on GABA_A_RS clustering may reflect the differences in the kinetics of TrkB activation (Ji et al., 2010) and may contribute to the homeostatic regulation of GABAergic synaptic strength (Tyagarajan and Fritschy, 2010; Vlachos et al., 2013; Brady et al., 2018).

After its release into the synaptic cleft, the activity of GABA is terminated by the reuptake of the neurotransmitter, a process mediated by the GABA transporters (GATs). The surface expression of GABA transporter-1 (GAT-1), the major GABA transporter expressed by both neurons and astrocytes (Guastella et al., 1990), is upregulated in neuronal cells by BDNF-mediated tyrosine kinase-dependent phosphorylation (Law et al., 2000; Whitworth and Quick, 2001). However, the neurotrophin was found to inhibit GAT-1-mediated GABA transport at the isolated nerve endings (Vaz et al., 2008), suggesting that this effect is very localized, to delay GABA uptake by the nerve terminal, thereby enhancing synaptic actions of GABA. In contrast with the effects at the synapse, BDNF may accelerate the uptake of GABA at extrasynaptic sites, allowing replenishment of neuronal pools of GABA. Furthermore, BDNF enhances GABA transport in rat cortical astrocytes by modulating the trafficking of GAT-1 from the plasma membrane (Vaz et al., 2011).

BDNF also regulates genes transcription of GABA_A_R subunits (Bell-Horner et al., 2006) GAD65 (Sánchez-Huertas and Rico, 2011) and GATs (Vaz et al., 2011), through the recruitment of the ERK-MAP kinase cascade, which activates the cAMP-response element (CRE)-binding protein (CREB; Figure 1; Yoshii and Constantine-Paton, 2010). In an opposite way, the downstream signaling pathway triggered by proBDNF/p75^NTR^ activates the JAK-STAT pathway leading to the induction of the cAMP early repressor ICER, which mediates the downregulation of GABA_A_Rs ß3 gene synthesis (Figure 1). Interestingly, the activation of this pathway precedes the decrease of GABA_A_Rs ß3 cell surface expression (Riffault et al., 2014).

Other reports have also suggested that in rat visual cortex and cerebellar Purkinje cells, the BDNF/TrkB signaling modulates GABA_A_Rs mediated currents through the PLCγ-Ca^2 +^ and CaMK pathways (Cheng and Yeh, 2003; Mizoguchi et al., 2003). In immature cultured rat hippocampal and hypothalamic neurons, the BDNF/TrkB dependent increase in GABA_A_Rs plasma membrane expression occurs when activation of GABA_A_Rs lead to a depolarization of the membrane potential, which in turn triggers the release of BDNF (Obrietan et al., 2002; Porcher et al., 2011). In more mature cultured rat hippocampal neurons and murine cerebellar granule cells, BDNF decreases the plasma membrane expression of GABA_A_Rs (Brünig et al., 2001; Cheng and Yeh, 2003). In parallel, BDNF/trkB signaling reduces the excitability of parvalbumin-positive interneurons in the mouse dentate gyrus (Holm et al., 2009). Surprisingly, these neurons do not express the proBDNF receptor p75^NTR^ (Dougherty and Milner, 1999; Holm et al., 2009). The change in the regulation of GABA_A_Rs cell surface expression by BDNF coincides with a shift in GABA polarity (depolarization to hyperpolarization), attributed to the activity of KCC2 (Rivera et al., 1999) which is also regulated by both forms of BDNF. A recent study showed that increased proBDNF/p75^NTR^ signaling disrupts the developmental GABAergic sequence by maintaining a depolarizing GABA response in a KCC2-dependent manner in mature cortical neurons (Riffault et al., 2018). In developing neurons, BDNF increases KCC2 expression on the level of mRNA transcription (Aguado et al., 2003; Rivera et al., 2004; Ludwig et al., 2011). In line with these observations, it was shown that the expression of KCC2 is significantly decreased in *trkB^−/–^* mice hippocampi (Carmona et al., 2006) whereas, in adult neurons BDNF decreases both mRNA and protein KCC2 (Rivera et al., 2002, 2004; Wake et al., 2007; Shulga et al., 2008; Boulenguez et al., 2010). In accordance with these results, neurons in the dorsal horn of the spinal cord treated with BDNF showed a depolarizing shift of the GABA reversal potential (Coull et al., 2003, 2005). The actions of BDNF/TrkB signaling on GABAergic synapses are developmentally regulated, with BDNF leading to an increase of KCC2 expression in immature neurons through activation of Shc pathway, and a decrease in adult neurons through activation of both Shc and PLCγ cascades (Rivera et al., 2002, 2004; Figure 1).

Altogether, these findings suggest that the relative availability of the two forms of BDNF, pro and mature, could affect the excitatory/inhibitory balance during the development by regulating the polarity and the synaptic strength of GABAergic transmission.

## GABA_B_R and BDNF Interplay

Similarly to BDNF, a crucial factor regulating the development of inhibitory transmission is GABA itself (Ben-Ari et al., 2007; Gaiarsa et al., 2011). In the neocortex, extracellular GABA signaling regulates the development of GABAergic inhibition through GABA_A_ and GABA_B_ receptors. During the developmental period, ambient GABA may also participate in neuronal network construction and synaptogenesis. In the visual cortex of mice, Chattopadhyaya et al. (2007) demonstrated that the tonic activation of GABA_A_ and GABA_B_ receptors regulates the axonal branching of basket-cell interneurons. They reported that reducing GABA levels in a single basket cell results in a decrease of perisomatic GABAergic inputs on the pyramidal cells. This deficit of synaptic transmission is partially restored by GABA uptake blocker or GABA_A_ and GABA_B_ receptor agonists. In agreement with this study, knockout of the GABA_B1_ subunit leads to altered maturation of GABAergic synaptic transmission in murine hippocampal neurons and synaptic activation of GABA_B_Rs promotes the development of GABAergic synapses (Fiorentino et al., 2009). The mechanisms are not fully understood but may likely involve the BDNF/TrkB signaling. Indeed, the trophic action of GABA_B_Rs was prevented by BDNF scavenger (TRkB-IgG) and not observed in BDNF KO mice (Fiorentino et al., 2009). Moreover, the stimulation of GABA_B_Rs induce a calcium-dependent release of BDNF via the PLC-PKC signaling cascade and L-type voltage-gated calcium channels (Fiorentino et al., 2009; Kuczewski et al., 2011). Finally, in the developing rat hippocampus, it was shown that activation of GABA_B_Rs also increased the phosphorylation levels of the α-CamKII, which play a critical role in BDNF release (Fischer et al., 2005; Kolarow et al., 2007; Xu et al., 2008). Therefore, postsynaptic calcium increase and phosphorylation of α-CamKII may underlie the GABA_B_-R-mediated release of BDNF. Interestingly, the regulated secretion of BDNF following GABA_B_ receptor activation increases the number of GABA_A_ ß2/3 subunits receptors at the postsynaptic membrane (Kuczewski et al., 2011). Thus, the interplay between GABA_B_Rs activation and the subsequent BDNF secretion in developing hippocampal neurons contribute to the functional maturation of GABAergic synaptic transmission.

## BDNF and GABA Interplay in Epilepsy

**Figure 2:**
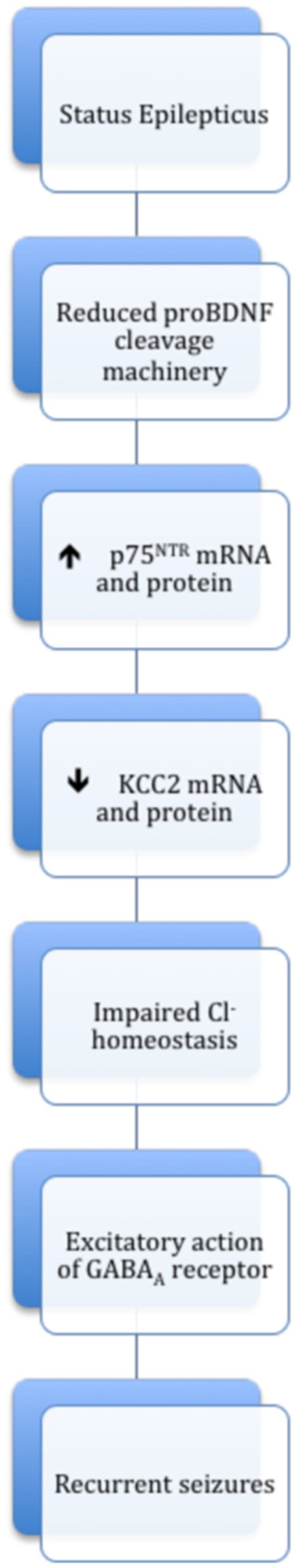
Scheme summarizing the causal relationship between proBDNF/p75^NTR^ and depolarizing action of GABA during epileptogenesis. Elevated amounts of proBDNF following status epilepticus (SE) are associated with reduced proBDNF cleavage machinery and increased expression of p75^NTR^. The proBDNF/p75NTR response donwregulates KCC2, which promotes a chloride homeostasis dysregulation leading to an excitatory action of GABA and facilitate recurrent seizures.

Epilepsy is a brain disorder characterized by the appearance of spontaneous recurrent seizures due to network hyperexcitability (Fischer et al., 2005). Neurotrophic signaling pathways are over-activated after status epilepticus (SE) and seem to contribute to epileptogenesis by promoting neuronal cell deaths and rewiring of excitatory networks (Koyama et al., 2004; Unsain et al., 2008; Goldberg and Coulter, 2013). Similarly, changes in GABAergic neurotransmission and altered neuronal Cl^−^ homeostasis are considered to play a crucial role in epileptogenesis. Initial studies regarding the contribution of BDNF to epilepsy led to conflicting conclusions, with intrahippocampal BDNF perfusion or intraventricular injection of the BDNF scavenger TrkB-IgG, both being protective in a model of dorsal hippocampal kindling (Reibel et al., 2000; Binder et al., 2001). However, further studies reported that epileptogenesis was suppressed in mice with conditional deletion of TrkB in the brain (He et al., 2004) as well as in mice carrying a TrkB gene mutation that uncouples TrkB from the PLCγ (He et al., 2010). Interestingly, elevated levels of BDNF and TrkB following seizure activity or bath application of BDNF on hippocampal neurons trigger a down-regulation of KCC2 surface expression and a subsequent increase in neuronal excitability which most likely contributes to the establishment of recurrent seizures (Rivera et al., 2002; Wake et al., 2007). In addition to the pro-epileptogenic effect of mBDNF, it has been shown that proBDNF and p75^NTR^ are markedly increased after Pilocarpine-induced seizures. The elevated amounts of proBDNF following SE are associated with reduced proBDNF cleavage machinery that results from acute decreases in tPA/plasminogen proteolytic cascade and increases in API-1, an inhibitor of proBDNF cleavage (Reibel et al., 2000; Binder et al., 2001). Furthermore, two recent studies showed that proBDNF/p75^NTR^ response following SE selectively downregulates KCC2, which in turn promotes a chloride homeostasis dysregulation leading to an excitatory action of GABA_A_ receptors and facilitate epileptiform discharges (Kourdougli et al., 2017; Riffault et al., 2018; Figure 2). Interestingly, blockade of p75^NTR^ during the earliest phase of epileptogenesis restores KCC2 levels and reduces seizures frequency (Kourdougli et al., 2017; Riffault et al., 2018). These results suggest that proBDNF/p75^NTR^ play a critical role in the mechanisms of epileptogenesis (see Figure 2). It should be pointed, however, that apart from these pro-epileptogenic actions, BDNF could exert anti-epileptic effects (Paradiso et al., 2009; Bovolenta et al., 2010). Several observations support the view that at least part of the pro-epileptogenic actions of pro- or mature-BDNF relies on an alteration of GABAergic inhibition. Thus, although BDNF exerts beneficial effects on developing GABAergic synapses, exogenous applications of this neurotrophin decrease the efficacy of GABAergic inhibition on mature neurons (Berninger et al., 1995; Mizoguchi et al., 2003). In cultured hippocampal neurons, proBDNF promotes GABA_A_ receptor endocytosis and degradation (Riffault et al., 2014) and BDNF has been reported to reduce the probability of GABA release (Mizoguchi et al., 2003). At the transcriptional level, BDNF/TrkB signaling causes the repression of GABA_A_Rs α1 subunit gene through the activation of JAK-STAT pathway following SE (Lund et al., 2008). An important feature of epileptogenesis is a downregulation of KCC2 expression both in human epileptogenic tissues (Aronica et al., 2007; Huberfeld et al., 2007; Munakata et al., 2007; Shimizu-Okabe et al., 2011; Kahle et al., 2014) and in animal models of epilepsy (Jin et al., 2005; Kourdougli et al., 2017; Riffault et al., 2018). In patients with temporal lobe epilepsy, the decrease in KCC2 expression results in depolarizing GABAergic events in a minority of subicular pyramidal cells that contribute to inter-ictal like activity (Cohen et al., 2002; Huberfeld et al., 2007). These findings are consistent with reports of KCC2 downregulation and changes in the polarity of GABAergic response in animal models of epilepsy (Huberfeld et al., 2007; Barmashenko et al., 2011; Shimizu-Okabe et al., 2011; Kourdougli et al., 2017; Riffault et al., 2018). Because both forms of BDNF regulate the expression of KCC2 (Rivera et al., 1999; Ludwig et al., 2011), the decrease observed in epileptic tissues could be due to an imbalance between mBDNF/TrkB and proBDNF/p75^NTR^ signaling during the first postnatal weeks causing an impaired or delayed functional maturation of GABAergic inhibition. Alternatively, an excess of BDNF production and secretion associated with reductions in proBDNF cleavage in epileptic tissues (Ernfors et al., 1991; Thomas et al., 2016) could account for the decrease in KCC2 expression (Figure 2). Altogether, these findings show a complex picture in which BDNF signaling can influence the pathogenicity of epilepsy both ways. Further studies will be necessary to precise the role of the extracellular proBDNF/mBDNF ratio in GABAergic transmission during neuronal development and in different types of epilepsies.

Unveiling the mode of action of BDNF in the development and functioning of the GABAergic network is a promising quest for developing new cures of a number of neurological diseases. BDNF influences the development and functioning of the GABAergic network which in turn controls BDNF levels. As a result of this interaction, impairment of one of the two systems will most disturb the other, and since each of them is fundamental to normal CNS functioning, this will potentially lead to a host of neurological conditions. As of today, there is hope that investigation of the molecular pathways mediating the trophic action of BDNF may provide new insights into the normal development of the GABAergic network, providing new therapeutic strategies to improve the symptoms in a broad spectrum of GABA-related pathologies.

## Author Contributions

The review was conceptualized, written and edited by each of the authors. CP was the supervisor.

## Conflict of Interest Statement

The authors declare that the research was conducted in the absence of any commercial or financial relationships that could be construed as a potential conflict of interest.
